# 2q37 Deletions in Patients With an Albright Hereditary Osteodystrophy Phenotype and PTH Resistance

**DOI:** 10.3389/fendo.2019.00604

**Published:** 2019-08-29

**Authors:** Francesca Marta Elli, Luisa de Sanctis, Bruno Madeo, Maria A. Maffini, Paolo Bordogna, Arianna Pirelli, Maura Arosio, Giovanna Mantovani

**Affiliations:** ^1^Department of Clinical Sciences and Community Health, University of Milan, Milan, Italy; ^2^Department of Public Health and Pediatric Sciences, Regina Margherita Children's Hospital-AOU Città della Salute e della Scienza, University of Torino, Turin, Italy; ^3^Unit of Endocrinology, Department of Medical Specialties, Ospedale Civile di Baggiovara, Azienda Ospedaliero-Universitaria di Modena, Modena, Italy; ^4^Endocrinology Unit, Fondazione IRCCS Ca' Granda Ospedale Maggiore Policlinico, Milan, Italy

**Keywords:** pseudohypoparathyroidism, Albright hereditary osteodystrophy, brachydactyly-mental retardation syndrome, GNAS, 2q37 deletion

## Abstract

Pseudohypoparathyroidism (PHP) is a rare endocrine disorder derived from the defective activation of the cAMP pathway by the parathyroid hormone secondary to GNAS molecular defects. PHP subtypes are defined by the presence/absence of specific clinical/biochemical features. PHP1A is characterized by resistance to multiple hormones with features of Albright hereditary osteodystrophy (AHO), while pseudopseudohypoparathyroidism (PPHP) is characterized by AHO in the absence of PTH resistance. Small subsets of PHP and PPHP patients without known molecular defects have been re-diagnosed as being affected by the brachydactyly-mental retardation syndrome (BDMR), also known as the AHO-like syndrome. This study aimed to analyse 24 PHP1A and 51 PPHP patients without a molecular diagnosis for the presence of BDMR-associated 2q37 deletions to improve the differential diagnosis and to identify features that might help to avoid a misdiagnosis. Molecular investigations identified 4 deletions in 4 unrelated patients. The affected patients showed a combination of the most pathognomonic AHO features. Of note, 3 of the patients also displayed mild PTH resistance, and none of the patients developed ectopic ossifications. Our work confirmed the rarity of the misdiagnosis of BDMR in PHP patients through the identification of 4 patients bearing a 2q37 deletion in a cohort of 73 PHP patients (5.3%). Three patients with the deletion presented a PHP1A phenotype in the absence of any BDMR-specific findings. Further studies on larger case series are needed to elucidate the overlap between these clinical entities and to allow the early identification of patients.

## Introduction

Pseudohypoparathyroidism (PHP) is a rare endocrine disorder derived from the defective activation of the cAMP transduction pathway by the parathyroid hormone (PTH) secondary to molecular defects affecting the alpha subunit of the stimulatory G protein (Gsα), which is encoded by the *GNAS* gene (1). Since the first description in 1942, several PHP subtypes (PHP type 1A/PHP1A – MIM#103580, pseudopseudohypoparathyroidism/PPHP – MIM#612463, PHP type 1B/PHP1B-MIM#603233, PHP type 1C/PHP1C – MIM#612462, PHP type 2/PHP2 – MIM#203330 and progressive osseous heteroplasia POH – MIM#166350) have been defined by the presence/absence of specific clinical/biochemical features and by the presence/absence of underlying genetic, further divided into maternal or paternal, or epigenetic molecular GNAS defects (2–4). PHP1A is the clinical entity characterized by resistance to multiple hormones, mainly PTH and TSH, with features of Albright hereditary osteodystrophy (AHO) (3). PHP1A is associated with *GNAS* genetic defects, both point mutations and deletions, on the maternal allele. PPHP is defined as AHO in the absence of PTH resistance and is associated with paternal GNAS genetic alterations (5).

Research studies on these diseases allowed the identification of both clinical and molecular overlap among subtypes and with closely related disorders deriving from alterations of elements involved in the cAMP transduction pathway other than the Gsα, making the classification obsolete (4, 6). A novel nomenclature and classification including all disorders of the PTH/PTHrP receptor signaling pathway have been recently proposed, and the newly coined name is inactivating PTH/PTHrP signaling disorders (iPPSDs) (7). One of the additional strengths of the new classification is the provision of a category, iPPSDx, for those patients lacking a known genetic/epigenetic molecular determinant (8).

In past years, small subsets of clinically defined PHP and PPHP patients without known molecular defects have been re-diagnosed as being affected by the brachydactyly-mental retardation syndrome (BDMR, also known as the AHO-like syndrome or the 2q37 microdeletion syndrome, MIM#600430), as they carried deletions with breakpoint at or within chromosome 2 region q37 (9).

The BDMR syndrome is characterized by a spectrum of clinical features with different penetrance, including the AHO-like phenotype, with mild-moderate intellectual/developmental/ behavioral abnormalities, short stature, obesity, characteristic facies and brachydactyly type E (10).

In the present study, we screened 24 PHP1A and 51 PPHP patients without known genetic and epigenetic defects (hereafter, patients will be referred to as iPPSDx according to the novel proposed classification) for the presence of BDMR-associated 2q37 deletions to improve the differential diagnosis of iPPSDs and to identify features that might help in avoiding misdiagnoses.

## Materials and Methods

The study included 73 iPPSDx patients, defined as patients with a previous clinical diagnosis of PHP1A (*n* = 24) and/or PPHP (*n* = 49) without known molecular defects.

According to the recent classification of iPPSDs, the clinical and biochemical major criteria for PHP and related disorders are PTH resistance, and/or subcutaneous ossifications, and/or brachydactyly type E. The minor criteria include TSH resistance, other hormonal resistances, motor or cognitive impairment, intrauterine and/or postnatal growth retardation, obesity/overweight and flat nasal bridge and/or maxillary hypoplasia and/or round face. Brachydactyly type E is associated with other diseases and must be combined with at least one more major or 2 minor criteria to establish the diagnosis of iPPSD (7, 8).

Among our whole series of iPPSDx patients, in the present study, we selected patients presenting with at least 2 major criteria or 1 major criterion plus at least 2 minor criteria. Therefore, we selected those patients with major signs of AHO with or without hormone resistance. The clinical details of the investigated cohort are summarized in Supplementary Table 1. All the subjects involved in the study gave informed consent for genetic and epigenetic studies.

Previous investigation of patient's genomic DNA samples extracted from peripheral blood (Nucleon BACC2 genomic DNA purification kit, GE Healthcare, Piscataway, NJ, USA) by direct sequencing of the *GNAS, PRKAR1A* and *PDE4D* coding exons and flanking intronic sequences (ENSEMBL reference sequence ENSG00000087460, ENSG00000108946, and ENSG00000113448, respectively) and *PDE3A* exon 4 (ENST00000359062), as well as the methylation specific-multiplex ligand-dependent probe amplification (MS-MLPA) of the GNAS locus (ME031 GNAS probemix by MRC-Holland, Amsterdam, The Netherlands) did not reveal any (epi)genetic abnormality associated with iPPSDs (11–13).

The presence of 2q37 deletions was assessed by MLPA using the P264-Human Telomere-9 (MRC-Holland, Amsterdam, The Netherlands). MS-MLPA and MLPA data analyses were performed by the Coffalyser software (MRC-Holland, Amsterdam, The Netherlands).

The 2q37 variable number tandem repeats (VNTRs) genotyping was used to confirm the deletions found by MLPA. Briefly, genetic markers were PCR-amplified, and after capillary electrophoresis on the ABI3130xl Genetic Analyser, the peak pattern was evaluated with the Peak Scanner software (Applied Biosystems, Foster City, CA). We used a three-primer approach based on the simultaneous use of a couple of sequence-specific primers associated with a fluorescently labeled universal forward M13 tailed FAM oligonucleotide. All the primers and experimental conditions are available upon request.

## Results

The MLPA analysis of the 2q37 region identified 4 deletions in 4 unrelated patients. The assay showed a 3 Mb deletion from the *HDAC4* gene to the *PDCD1* gene in patient 2, a 3,5-4,5 Mb deletion from the *TRAF3IP1* gene to the *PDCD1* gene in patients 3 and 4, and a 5-8 Mb deletion from the *COL6A3* gene to the *PDCD1* gene in patient 1 (Supplementary Figure 1 and Supplementary Table 2).

Although we were not able to identify the precise location of the breakpoints, the analysis of VNTRs in the 2q37 region allowed the approximate delineation of the extension of deletions because heterozygous markers represented a biallelic condition. In particular, the deletion extension range and the number of deleted genes were: 2′924′948-3′893′175 bps/38-40 genes for patient 2, 3′549 ′580-4′005′555 bps/38-39 genes for patient 3, 3′893′241-4′526′825 bps/41-51 genes for patient 4 and 4′902′697-8′798′972 bps/53-54 genes for patient 1 (Supplementary Figure 1 and Supplementary Table 2). Unfortunately, biological samples from the parents were not available; thus, we were not able to determine the inheritance pattern of defects (inherited vs. *de novo*) or the affected allele (maternal vs. paternal).

**Figure 1:**
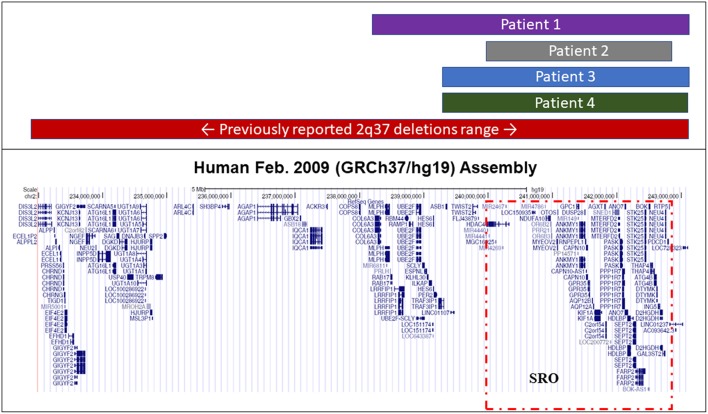
Extension of known 2q37 deletions. Representative figure from the UCSC Genome Browser summarizing the extension range of previously reported BDMR-associated and novel deletions found in our iPPSDx patients. The smallest region of overlap (SRO) among our deletions, corresponding to the one discovered in patient 2, is highlighted in red.

The smallest region of overlap (SRO) containing the gene or genes potentially involved in the clinical presentation, that is the region ranging from nucleotides 239′306′199 to 242'855′711, where all four found deletions overlapped, removed ~3.5 Mbp hosting 38 genes (Figure 1 and Supplementary Table 2).

The clinical details of patients with deletions are summarized in Table 1. Of note, all patients with deletions displayed brachydactyly and cognitive impairment, and 3 of these patients also displayed mild resistance to PTH, extremely elevated phosphate levels and exhibited 25-OH vitamin D levels within the normal range.

**Table 1:** Clinical and biochemical characteristics of patients with deletions.

		**Patient 1**	**Patient 2**	**Patient 3**	**Patient 4**
Clinical and auxological data	Age range at clinical evaluation	10–15 y	10–15 y	10–15 y	20–25 y
	Pre- and perinatal growth retardation	IUGR	AGA	AGA	AGA
	Postnatal growth retardation	NO	YES	NO	NO
	Obesity/overweight	YES (75°centile)	NO (<3°centile)	YES (>97°centile/ BMI = 28.3 at 15 y)	YES (BMI = 31)
	Brachydactyly	YES (brachymetacarpia)	YES (brachydactyly - brachymetacarpia - brachymetatarsia)	YES (brachymetacarpia - brachymetatarsia)	YES (brachymetacarpia)
	Mental retardation	YES	YES	YES	YES
	Facial dysmorphisms	NO	YES (prominent forehead, microphthalmia, rotated posteriorly auricles, small nose, short filter, wide mouth)	NO	YES
	Additional features	slightly increased bone age (13$\frac{1}{2}$-14 y vs. 12 y of chronological age)	Legs exostosis - 3 accidental fractures (ulna and wrist) - elbow dislocation due to joint laxity	febrile convulsions after 1 y	phimosis, genu valgum, scoliosis, bilateral flat feet
	PTH	↑	↑↑	↑	↔
		(135 pg/mL)	(130 pg/mL)	(83 pg/mL)	(49 pg/mL)
Biochemical data	Ca	↓	↓	↓	↔
		8.82 mg/dL	7.97 mg/dL	7.9 mg/dL	9.38 mg/dL
	P	↑	↑	↑	↑
		4.46 mg/dL	12.4 mg/dL	13.33 mg/dL	13.33 mg/dL
	TSH	↔	↔	↔	↔
		2.09 mUI/L	2.63 mUI/L	0.7 mUI/L	

*IUGR, intrauterine growth retardation; y, years; AGA, appropriate for gestational age. Normal range: PTH 10–65 pg/mL; Ca 9–10.5 mg/dL; P 2.8–4.5 mmol/dL; TSH 0.4–3.9 mUI/L*.

## Discussion

Pseudohypoparathyroidism demonstrated much overlap from the clinical point of view, with diseases in the differential diagnosis, and currently, it is often difficult to make a conclusive diagnosis without a molecular confirmation of the underlying genetic defect (1).

Despite the high detection rate of molecular defects, a percentage of patients with a clinical suspicion of PHP still lack a confirming diagnosis; thus, there is a need to screen for alterations associated with diseases sharing a clinical similarity. Recently, deletions of chromosome 2q37.2 have been detected in a small subset of patients with PHP but without *GNAS* defects, further confirming a phenotypic overlap with the BDMR syndrome (1, 9).

BDMR is a complex syndrome with a significantly variable presentation of characteristic dysmorphic features, as patients show a range of severity of physical findings, mental retardation and behavioral characteristics. Despite the dysmorphisms, malformations, additional late-onset abnormalities and neurodevelopmental/behavioral traits summarized in Table 2, BDMR was initially named as the “AHO-like syndrome.” The hallmarks for diagnosis of BDMR include brachymetaphalangia (short fourth and fifth metacarpals and metatarsals), early developmental delay or frank mental retardation (from mild to severe), short stature (sometimes early overgrowth with subsequent premature closure of the epiphyses and short final height) and obesity (10).

**Table 2:** Table summarizing features associated with the BDMR syndrome.

**ADDITIONAL FEATURES ASSOCIATED WITH THE BDMR PHENOTYPE**
**Typical facial dysmorphisms (most cases)** Prominent forehead (pt 2) Arched eyebrows Upslanting palpebral fissures Midface hypoplasia (pt 2) Depressed nasal bridge (pt 2) Thin upper lip (pt 2) Anteverted lobules (pt 2)
**Major malformations (~30% of pts)** Congenital heart malformations (i.e., ventricular septal defects, aortic coarctation or hypoplasia) Gastrointestinal and renal anomalies (i.e., pyloric stenosis, duodenal or esophageal atresia) Genitourinary malformations (i.e., horseshoe kidney, hypospadias, hypoplastic gonads, bifid uterus and undescended testes) Central nervous system malformations (i.e., hydrocephaly, holoprosencephaly) Congenital skeletal malformations (i.e., hip dislocation, fused cervical vertebrae, fractures, arched or cleft palate) (pt 2)
**Other features and late-onset abnormalities** Sparse or thin hair Eczema Recurrent otitis media Sinusitis and lower respiratory infections Joint laxity (pt 2) Umbilical and inguinal hernias Articulation dislocation (pt 2)
**Neurodevelopmental and behavioral traits (frequently reported)** Hypotonia improving with time Seizure disorder unrelated to brain malformation Epilepsy Complex febrile seizures (pt 3) Autistic features (i.e., repetitive behaviors, a deficit in communication and social interaction, stereotypic movements, intermittent aggression, hyperactivity, attention deficit, obsessive-compulsive disorder and sleep disturbances) (pt 1 & 3)

*Features observed in our patients with deletions are reported by the patient's number in brackets*.

In the present study, we investigated the presence of 2q37 deletions in our cohort of 73 iPPSDx patients, in whom relevant differential diagnoses had been excluded. Molecular analyses identified 4 different overlapping 2q37 deletions, ranging from approximately 3 Mbp to 8 Mbp extensions, in 4 unrelated patients.

The detected deletions completely overlapped with previously reported 2q37 BDMR-associated abnormalities, and the approximately 3.5 Mbp smallest region of overlap hosted 38 genes already proposed as being related to the AHO-like phenotype (*GCP1, GPR35, HDAC4, CAPN10, HDLBP, PASK, FARP2*, and *STK25*) (10, 14–18). Our data further supported the hypothesis that one or more of these genes are important for skeletal morphogenesis and neurodevelopment and that their gene products could interact with Gsα-mediated transduction pathways without affecting those activated by other mediators.

The common clinical features shared by our patients with deletions were brachydactyly and mental retardation, without thyroid dysfunction and ectopic ossifications.

Interestingly, none of these patients developed ectopic ossifications; therefore, extraskeletal bone formation should remain to be considered as a typical feature associated with iPPSDs only (and particularly with GNAS defects).

When we looked for additional BDMR-specific physical findings, we found that patient 2 presented congenital skeletal malformations, including leg exostosis, frequent accidental fractures and joint laxity. Patient 3 suffered from febrile convulsions after the first year, while patient 4 showed skeletal malformations, including scoliosis and bilateral flat feet. To the best of our knowledge, no patients, other than patient 2 with exostosis, have been reported until now, and we did not analyse patient 2 for the presence of an additional genetic defect, in particular for *EXT1* or *EXT2* mutations.

When comparing patients with deletions with the whole cohort of iPPSDx patients, we did not find gene-specific clinical manifestations that helped to define patient-specific algorithms to properly address the genetic testing. Patients with deletions showed clinical signs and symptoms, both common and uncommon, to PHP and BDMR.

Recently, we investigated the presence of 2q37 deletions in our cohort of PHP-1B/iPPSD3 patients, and we discovered the first 2 cases of patients affected by both GNAS sporadic imprinting defects and BDMR-associated deletions (19). Although the deletions identified in the two studies are slightly different in extension, the deletions all overlap and determine the loss of genes starting from *HDAC4*. Moreover, from a clinical point of view, we observed a certain similarity with the patients described in the present paper, as iPPSD3 patients showed PTH resistance and signs of AHO. Moreover, iPPSD3 patients were affected by additional features not typical of PHP: strabismus, gastroesophageal and vesicoureteral reflux, joint subluxation and scoliosis (19). These data make it even more difficult to distinguish PHP from BDMR by only evaluating clinical features. Although additional studies are needed to confirm and further characterize the clinical specific presentations and to identify candidate genes, our results might explain the presence of non-typical signs in a subgroup of PHP patients.

In the present paper, three patients with deletions showed a mild resistance to PTH, brachydactyly and mental and/or behavioral defects; thus, we further examined a cluster of 11 patients presenting those three features, but we did not observe any noteworthy phenotypic differences. To note that all deleted patients, even the one with normal PTH levels, suffered from hyperphosphatemia.

Hormone resistance is typically absent in BDMR, while in PHP, PTH levels are variable but frequently severely elevated. To the best our knowledge, only one patient with an AHO-like phenotype and increased PTH levels has been described, and after additional biochemical investigations, the authors suggested a mild hormone resistance (20). Possible explanations are that, in published studies, the endocrine function was not systematically evaluated or that patients had not developed an overt hormone resistance at the moment of the evaluation.

2q37 deletions are inheritable defects with a 50% recurrence risk in the offspring; for genetic counseling purposes, patients and parents should be screened. Moreover, after the exclusion of defects affecting the Gsα-cAMP signaling pathway, screening for such defects should be considered in the evaluation of specific iPPSDx patients showing brachydactyly, mental and/or behavioral defects and, unexpectedly, elevated PTH serum levels.

In case of confirmation, patients should be re-evaluated and start a follow-up for BDMR, including echocardiogram, renal ultrasound for the possible development of renal cysts, periodic hearing screen for the risk of middle ear disease/dysfunction, and ophthalmologic evaluation. Obesity should be controlled, both in the child and the adult, and growth parameters should be monitored. To provide appropriate educational intervention, children should undergo an early developmental and behavioral assessment. Patients with joint laxity should practice low impact physical activities. The surveillance for Wilms' tumor should be performed in children with a breakpoint at or proximal to band 2q37.1. For additional common features, such as otitis, sinusitis, asthma and eczema, standard care should be provided (10).

In conclusion, with the present work, we confirmed the rarity of misdiagnosis of iPPSD in BDMR subjects, as we identified 4 deletions in 4 unrelated patients in the screening of 73 iPPSDx patients (5.3%). All patients with deletions presented brachydactyly and mental retardation in the absence of thyroid dysfunction and ectopic ossifications, while three of these patients also exhibited increased PTH levels. We did not find any additional BDMR-specific physical findings that might help in identifying patients requiring a molecular investigation for terminal 2q37 deletions. Further studies are needed to elucidate the clinical overlap between PHP and BDMR and to formulate new classification algorithms for the early identification of patients.

## Data Availability

All datasets for this study are included in the manuscript and the Supplementary Files.

## Ethics Statement

Informed consent was obtained from all the patients (or from legal guardians for minors) and relatives included in the present study. All the procedures were performed in compliance with relevant legislation and institutional guidelines and were approved by the IRCCS Fondazione Cà Granda Ospedale Maggiore Policlinico institutional committee.

## Author Contributions

FE conceived and designed the project, analyzed and interpreted data, and was a major contributor in writing the manuscript. GM conceived and designed the project, followed patients, interpreted data, and was a major contributor in writing the manuscript. MM, AP, and PB acquired and analyzed data. LdS, BM, and MA followed patients and were minor contributors in writing the manuscript. All authors read and approved the final manuscript.

### Conflict of Interest Statement

The authors declare that the research was conducted in the absence of any commercial or financial relationships that could be construed as a potential conflict of interest.
