# Supplementary material for: 2q37 Deletions in Patients With an Albright Hereditary Osteodystrophy Phenotype and PTH Resistance
Source: Front Endocrinol (Lausanne). 2019 Aug 29;10:604. doi: 10.3389/fendo.2019.00604 (PMC6727065; doi:10.3389/fendo.2019.00604)
Supplement: Supplementary file 1 [file Data_Sheet_1.PDF]

Supplementary Figure 1. Panel A: representative MLPA electropherogram obtained in a normal control. MLPA probes, according to their amplicon lenght, are reported on the X-axis. The Y-axis reports the relative fluorescence units (RFU). Panel B: Plot of the MLPA analysis by Coffalyser of a normal control. MLPA probes, grouped according to the chromosomal location, are reported on the X-axis. The Y-axis reports the calculated probe ratio that allows to predict the presence of copy number variations (CNVs). In particular, yellow Xs and green boxes are mean ratios and the 95% confidence interval over the reference samples for each probe. Blue and red lines delineate the range of ration values obtained in case of a normal biallelic condition. Error bars represent the 95% confidence interval of a probe that surround the calculated probe ratio, which is the black dot. Panels C-F: Plots of the MLPA analysis by Coffalyser showing the 2q37 deletions found in our cohort of iPPSDx patients (C = patient 1; D = patient 2; E = patient 3; F = patient 4). Red dots point out the presence of the deletion of one allele (probe ratio = 0.5).

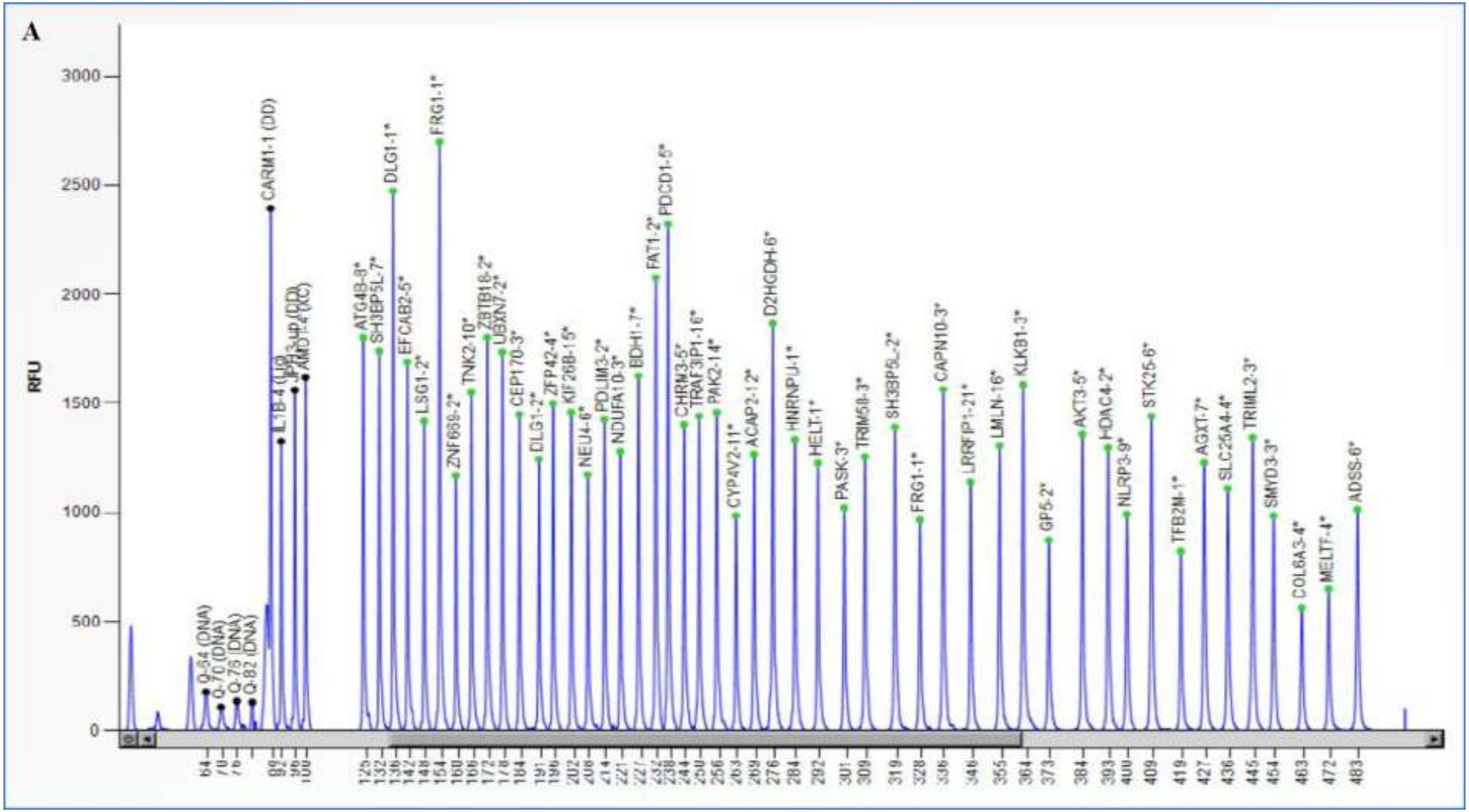
A
RFU
3000
2500
2000
1500
1000
500
0
Q-64 (DNA)
Q-70 (DNA)
Q-76 (DNA)
Q-82 (DNA)
CARM1-1 (DD)
ATG4B-8*
SH3BP5L-7*
DLG1-1*
EFCAB2-5*
LSG1-2*
FRG1-1*
ZNF668-2*
TNK2-10*
ZBTB18-2*
UBXN7-2*
CEP170-3*
DLG1-2*
ZFP42-4*
KIF26B-15*
NEU4-6*
PDLIM3-2*
NDUFA10-3*
BDH1-7*
FAT1-2*
PDCD1-5*
CHRM3-5*
TRAF3IP1-16*
PAK2-14*
CYP4V2-11*
ACAP2-12*
D2HGDH-6*
HNRNPU-1*
HELT-1*
PASK-3*
TRIM58-3*
SH3BP5L-2*
FRG1-1*
CAPN10-3*
LRRFIP1-21*
LMLN-16*
KLKB1-3*
GP5-2*
AKT3-5*
HDAC4-2*
NLRP3-9*
STK25-6*
TFB2M-1*
AGXT-7*
SLC25A4-4*
TRIML2-3*
SMYD3-3*
COL6A3-4*
MELTF-4*
ADSS-6*
64
125 132 136 142 148 154 160 166 172 178 184 191 196 202 208 214 221 227 232 238 244 250 256 263 269 276 284 292 301 309 319 328 336 346 355 364 373 384 393 400 409 419 427 436 445 454 463 472 483

B

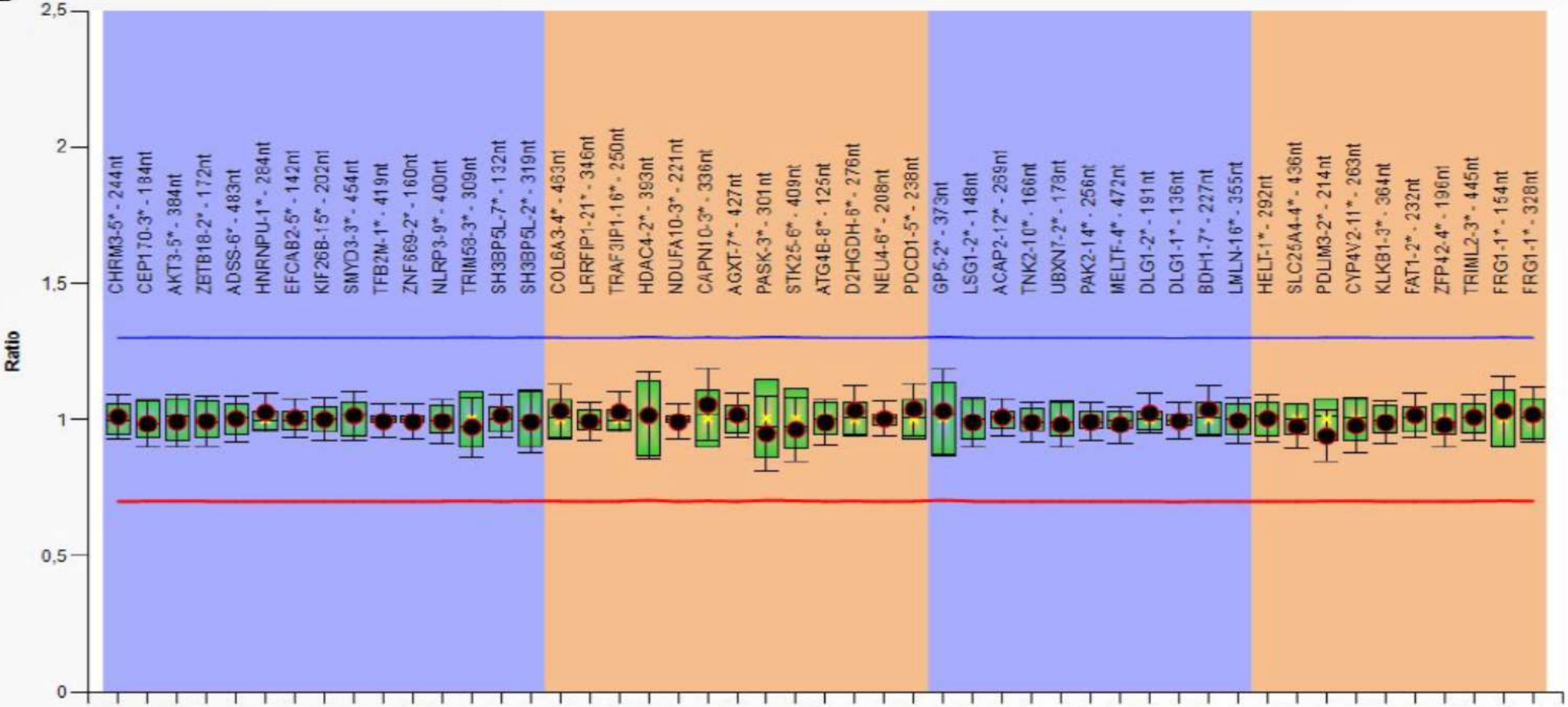

Ratio
2,5
2
1,5
1
0,5
0
CHRM3-5* - 244nt
CEP170-3* - 184nt
AKT3-5* - 384nt
ZBTB18-2* - 172nt
ADSS-6* - 483nt
HNRNPU-1* - 284nt
EFCAB2-5* - 142nt
KIF26B-15* - 202nt
SMYD3-3* - 454nt
TFB2M-1* - 419nt
ZNF669-2* - 160nt
NLRP3-9* - 400nt
TRIM58-3* - 309nt
SH3BP5L-7* - 132nt
SH3BP5L-2* - 319nt
COL6A3-4* - 463nt
LRRFIP1-21* - 346nt
TRAF3IP1-16* - 250nt
HDAC4-2* - 393nt
NDUFA10-3* - 221nt
CAPN10-3* - 336nt
AGXT-7* - 427nt
PASK-3* - 301nt
STK25-6* - 409nt
ATG4B-8* - 125nt
D2HGDH-6* - 276nt
NEU4-6* - 208nt
PDCD1-5* - 238nt
GP5-2* - 373nt
LSG1-2* - 148nt
ACAP2-12* - 269nt
TNK2-10* - 166nt
UBXN7-2* - 178nt
PAK2-14* - 256nt
MELTF-4* - 472nt
DLG1-2* - 191nt
DLG1-1* - 136nt
BDH1-7* - 227nt
LMLN-16* - 355nt
HELT-1* - 292nt
SLC25A4-4* - 436nt
PDLIM3-2* - 214nt
CYP4V2-11* - 263nt
KLKB1-3* - 364nt
FAT1-2* - 232nt
ZFP42-4* - 196nt
TRIML2-3* - 445nt
FRG1-1* - 154nt
FRG1-1* - 328nt

C

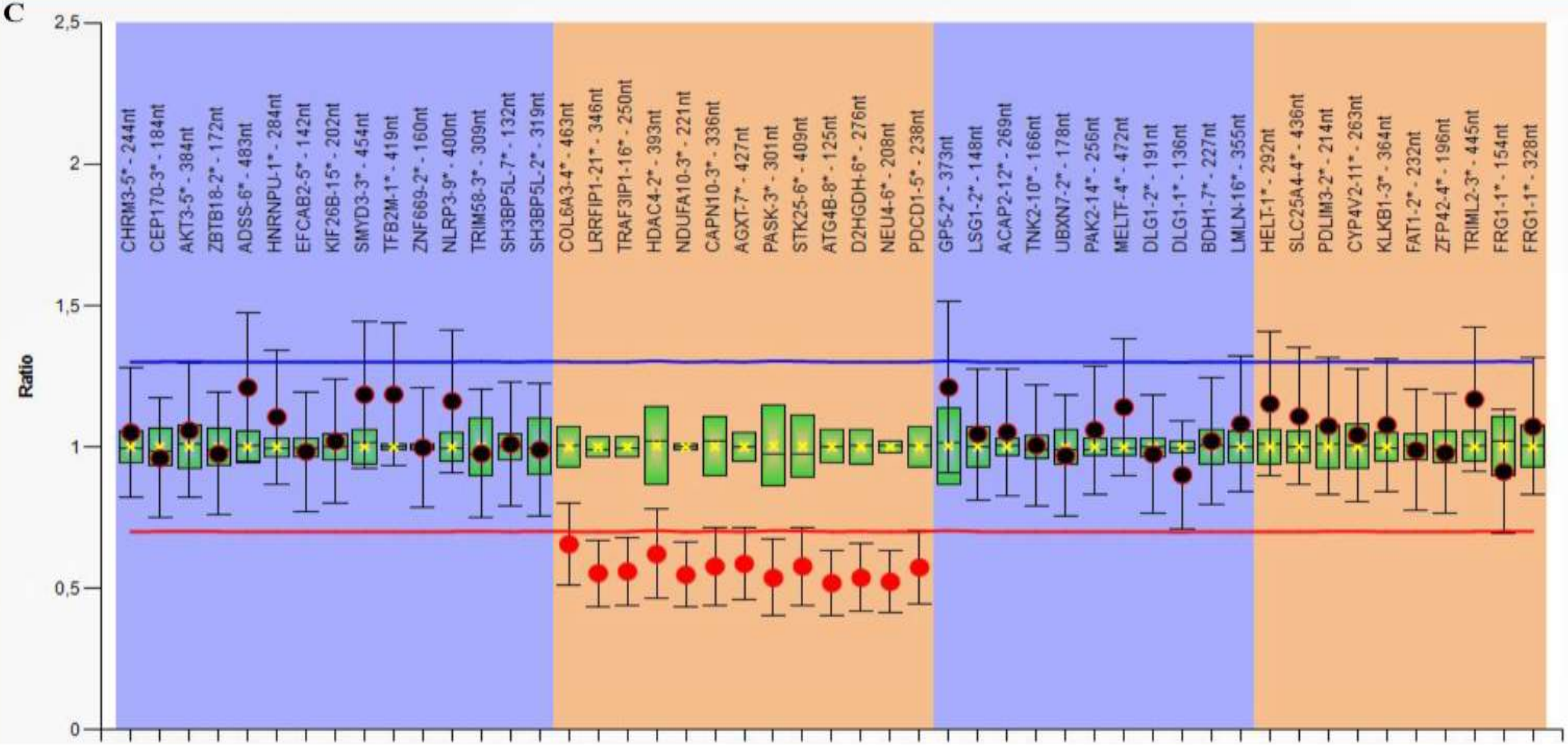
2,5
2
1,5
1
0,5
0
Ratio
CHRM3-5* - 244nt
CEP170-3* - 184nt
AKT3-5* - 384nt
ZBTB18-2* - 172nt
ADSS-6* - 483nt
HNRNPU-1* - 284nt
EFCAB2-5* - 142nt
KIF26B-15* - 202nt
SMYD3-3* - 454nt
TFB2M-1* - 419nt
ZNF669-2* - 160nt
NLRP3-9* - 400nt
TRIM58-3* - 309nt
SH3BP5L-7* - 132nt
SH3BP5L-2* - 319nt
COL6A3-4* - 463nt
LRRFIP1-21* - 346nt
TRAF3IP1-16* - 250nt
HDAC4-2* - 393nt
NDUFA10-3* - 221nt
CAPN10-3* - 336nt
AGXT-7* - 427nt
PASK-3* - 301nt
STK25-6* - 409nt
ATG4B-8* - 125nt
D2HGDH-6* - 276nt
NEU4-6* - 208nt
PDCD1-5* - 238nt
GP5-2* - 373nt
LSG1-2* - 148nt
ACAP2-12* - 269nt
TNK2-10* - 166nt
UBXN7-2* - 178nt
PAK2-14* - 256nt
MELTF-4* - 472nt
DLG1-2* - 191nt
DLG1-1* - 136nt
BDH1-7* - 227nt
LMLN-16* - 355nt
HELT-1* - 292nt
SLC25A4-4* - 436nt
PDLIM3-2* - 214nt
CYP4V2-11* - 263nt
KLKB1-3* - 364nt
FAT1-2* - 232nt
ZFP42-4* - 196nt
TRIML2-3* - 445nt
FRG1-1* - 154nt
FRG1-1* - 328nt

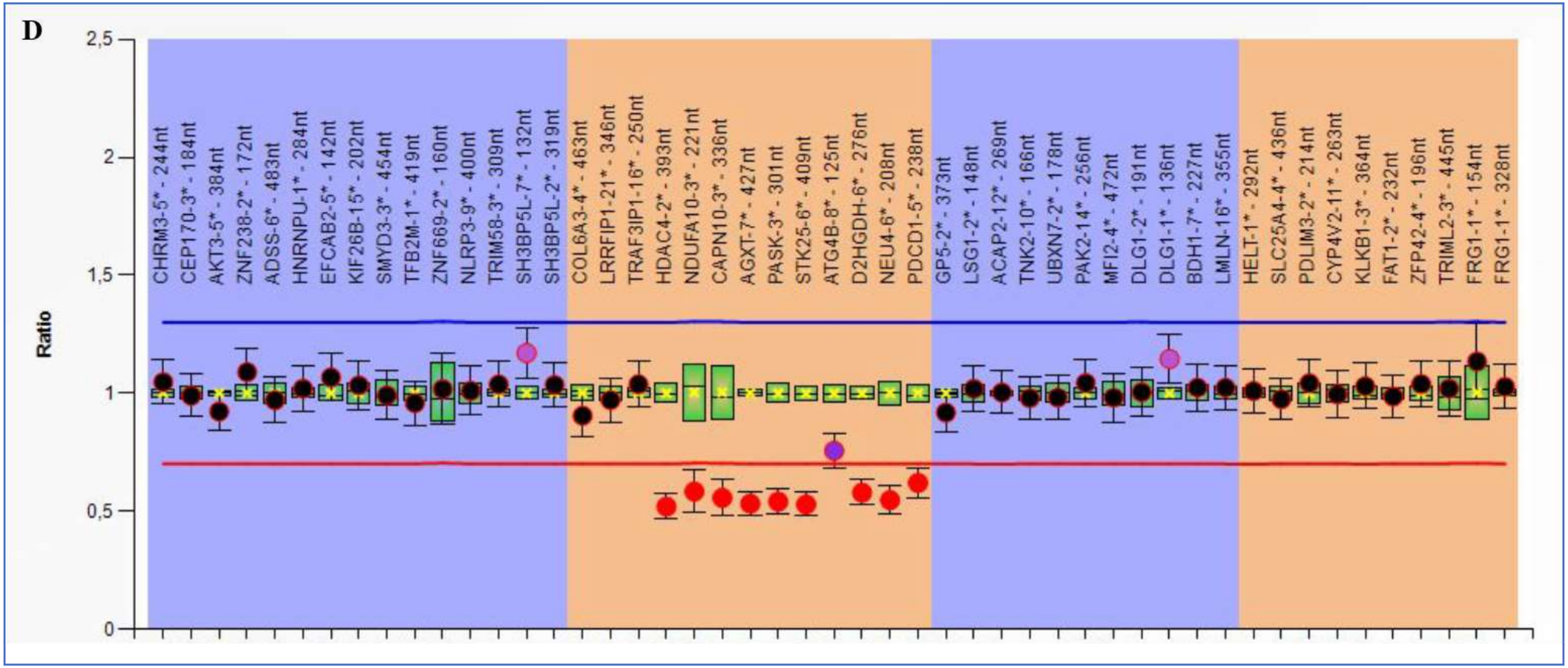
D
Ratio
2,5
2
1,5
1
0,5
0
CHRM3-5* - 244nt
CEP170-3* - 184nt
AKT3-5* - 384nt
ZNF238-2* - 172nt
ADSS-6* - 483nt
HNRNPU-1* - 284nt
EFCAB2-5* - 142nt
KIF26B-15* - 202nt
SMYD3-3* - 454nt
TFB2M-1* - 419nt
ZNF669-2* - 160nt
NLRP3-9* - 400nt
TRIM58-3* - 309nt
SH3BP5L-7* - 132nt
SH3BP5L-2* - 319nt
COL6A3-4* - 463nt
LRRFIP1-21* - 346nt
TRAF3IP1-16* - 250nt
HDAC4-2* - 393nt
NDUFA10-3* - 221nt
CAPN10-3* - 336nt
AGXT-7* - 427nt
PASK-3* - 301nt
STK25-6* - 409nt
ATG4B-8* - 125nt
D2HGDH-6* - 276nt
NEU4-6* - 208nt
PDCD1-5* - 238nt
GP5-2* - 373nt
LSG1-2* - 148nt
ACAP2-12* - 269nt
TNK2-10* - 166nt
UBXN7-2* - 178nt
PAK2-14* - 256nt
MFI2-4* - 472nt
DLG1-2* - 191nt
DLG1-1* - 136nt
BDH1-7* - 227nt
LMLN-16* - 355nt
HELT-1* - 292nt
SLC25A44-4* - 436nt
PDLIM3-2* - 214nt
CYP4V2-11* - 263nt
KLKB1-3* - 364nt
FAT1-2* - 232nt
ZFP42-4* - 196nt
TRIML2-3* - 445nt
FRG1-1* - 154nt
FRG1-1* - 328nt

E

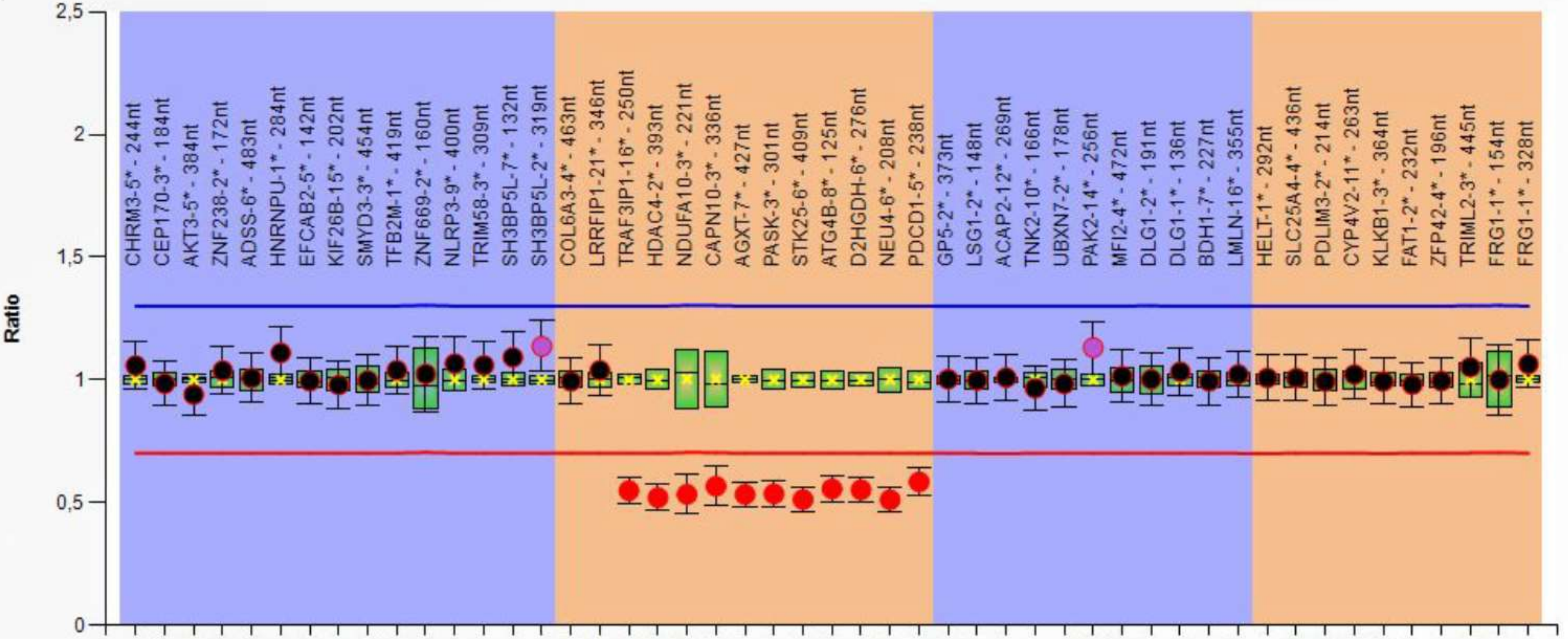
Ratio
2,5
2
1,5
1
0,5
0
CHRM3-5* - 244nt
CEP170-3* - 184nt
AKT3-5* - 384nt
ZNF238-2* - 172nt
ADSS-6* - 483nt
HNRNPU-1* - 284nt
EFCAB2-5* - 142nt
KIF26B-15* - 202nt
SMYD3-3* - 454nt
TFB2M-1* - 419nt
ZNF669-2* - 160nt
NLRP3-9* - 400nt
TRIM58-3* - 309nt
SH3BP5L-7* - 132nt
SH3BP5L-2* - 319nt
COL6A3-4* - 463nt
LRRFIP1-21* - 346nt
TRAF3IP1-16* - 250nt
HDAC4-2* - 393nt
NDUFA10-3* - 221nt
CAPN10-3* - 336nt
AGXT-7* - 427nt
PASK-3* - 301nt
STK25-6* - 409nt
ATG4B-8* - 125nt
D2HGDH-6* - 276nt
NEU4-6* - 208nt
PDCD1-5* - 238nt
GP5-2* - 373nt
LSG1-2* - 148nt
ACAP2-12* - 269nt
TNK2-10* - 166nt
UBXN7-2* - 178nt
PAK2-14* - 256nt
MFI2-4* - 472nt
DLG1-2* - 191nt
DLG1-1* - 136nt
BDH1-7* - 227nt
LMLN-16* - 355nt
HELT-1* - 292nt
SLC25A4-4* - 436nt
PDLIM3-2* - 214nt
CYP4V2-11* - 263nt
KLKB1-3* - 364nt
FAT1-2* - 232nt
ZFP42-4* - 196nt
TRIML2-3* - 445nt
FRG1-1* - 154nt
FRG1-1* - 328nt

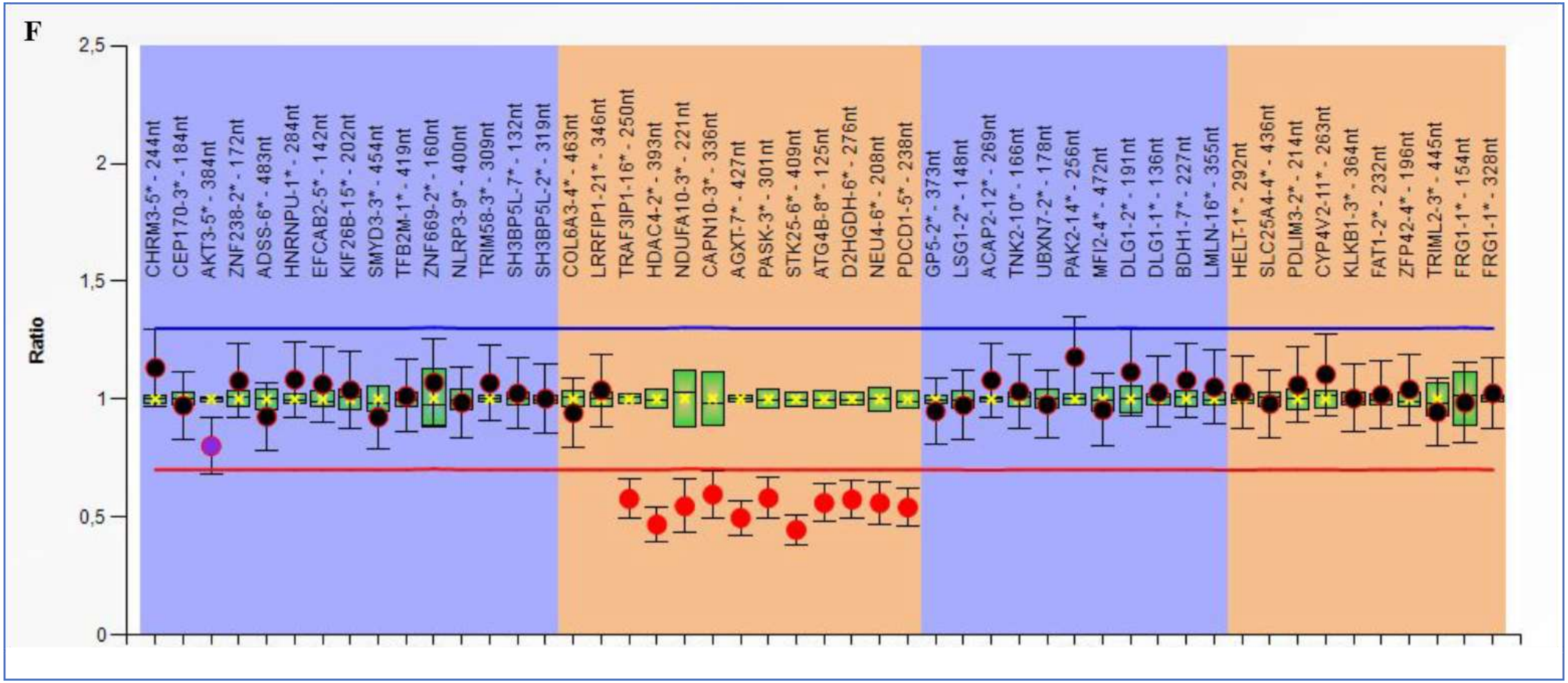
F
Ratio
2,5
2
1,5
1
0,5
0
CHRM3-5* - 244nt
CEP170-3* - 184nt
AKT3-5* - 384nt
ZNF238-2* - 172nt
ADSS-6* - 483nt
HNRNPU-1* - 284nt
EFCAB2-5* - 142nt
KIF26B-15* - 202nt
SMYD3-3* - 454nt
TFB2M-1* - 419nt
ZNF669-2* - 160nt
NLRP3-9* - 400nt
TRIM58-3* - 309nt
SH3BP5L-7* - 132nt
SH3BP5L-2* - 319nt
COL6A3-4* - 463nt
LRRFIP1-21* - 346nt
TRAF3IP1-16* - 250nt
HDAC4-2* - 393nt
NDUFA10-3* - 221nt
CAPN10-3* - 336nt
AGXT-7* - 427nt
PASK-3* - 301nt
STK25-6* - 409nt
ATG4B-8* - 125nt
D2HGDH-6* - 276nt
NEU4-6* - 208nt
PDCD1-5* - 238nt
GP5-2* - 373nt
LSG1-2* - 148nt
ACAP2-12* - 269nt
TNK2-10* - 166nt
UBXN7-2* - 178nt
PAK2-14* - 256nt
MFI2-4* - 472nt
DLG1-2* - 191nt
DLG1-1* - 136nt
BDH1-7* - 227nt
LMLN-16* - 355nt
HELT-1* - 292nt
SLC25A4-4* - 436nt
PDLIM3-2* - 214nt
CYP4V2-11* - 263nt
KLKB1-3* - 364nt
FAT1-2* - 232nt
ZFP42-4* - 196nt
TRIML2-3* - 445nt
FRG1-1* - 154nt
FRG1-1* - 328nt

Supplementary table 1. Clinical characteristics of patients included in the present study. Abbreviations and symbols: ID, identification number; F, female; M, male; rPTH, PTH resistance; BR, brachydactyly; OS, ectopic ossification; rTSH, TSH resistance; MR, mental retardation and/or behavioral defects; GR, intrauterine and/or postnatal growth retardation; OB, obesity; RF, round/dysmorfic facies; IUGR, intrauterine growth retardation. Patients bearing the 2q37 deletion are highlighted in bold.

PT ID	sex	clinical diagnosis	rPTH	BR	OS	n.major signs	rTSH	MR	GR	OB	RF	n.minor signs	additional features
**1**	**F**	**PHP**	**X**	**X**		**2**		**X**	**X**			**2**	**incrased bone age, IUGR, autoimmune thyroiditis**
**2**	**F**	**PHP**	**X**	**X**		**2**		**X**				**1**	**leg exostosis, accidental fractures, joint laxity**
**3**	**F**	**PHP**	**X**	**X**		**2**		**X**		**X**	**X**	**3**	
**4**	**M**	**PPHP**		**X**		**1**		**X**		**X**	**X**	**3**	**phimosis, genu valgum, scoliosis, bilateral flat feet**
**5**	M	PHP	X	X	X	3	X	X		X	X	4	
**6**	M	PHP	X	X		2		X	X		X	3	
**7**	F	PHP	X	X		2	X	X	X		X	4	
**8**	F	PHP	X	X		2		X	X	X	X	4	
**9**	F	PHP	X	X		2		X	X	X	X	4	
**10**	M	PHP	X	X		2		X		X		2	
**11**	M	PHP	X	X		2	X	X	X		X	4	
**12**	M	PHP	X	X	X	3			X	X	X	3	oligohydramnios, microcefalia, mild micrognathia, absence some permanent teeth, genu valgum, delayed bone age, osteoporosis
**13**	M	PHP	X	X	X	3			X			1	
**14**	F	PHP	X	X	X	3			X	X	X	3	
**15**	M	PHP	X	X		2	X		X			2	
**16**	F	PHP	X	X		2	X					1	
**17**	F	PHP	X	X		2			X		X	2	
**18**	F	PHP	X	X		2	X			X	X	3	
**19**	F	PHP	X	X		2			X			1	
**20**	M	PHP	X	X		2				X	X	2	
**21**	M	PHP	X	X		2	X			X	X	3	
**22**	M	PHP	X	X		2			X		X	2	
**23**	F	PHP	X	X		2				X	X	2	

**24**	F	PHP	X	X		2				X		1	
**25**	F	PPHP		X	X	2		X	X	X	X	4	
**26**	M	PPHP		X	X	2	X	X		X	X	4	
**27**	F	PPHP		X		1		X		X	X	3	
**28**	M	PPHP		X		1		X	X	X	X	4	
**29**	F	PPHP		X		1		X	X			2	
**30**	F	PPHP		X		1	X	X	X	X		4	
**31**	M	PPHP		X		1		X		X		2	
**32**	M	PPHP		X		1	X	X	X	X		4	hypospadias
**33**	M	PPHP		X		1		X	X	X	X	4	
**34**	F	PPHP		X		1	X	X	X	X	X	5	SGA, syndactyly, enamel hypoplasia, hallux valgus, dysmorphic femoral heads, excessive sweating, bilateral cyst wrist
**35**	F	PPHP		X		1		X	X	X		3	
**36**	F	PPHP		X		1		X		X	X	3	epilepsy, neonatal hypoglycemia
**37**	F	PPHP		X		1		X	X		X	3	
**38**	M	PPHP		X		1		X	X			2	
**39**	F	PPHP		X		1		X	X	X	X	4	
**40**	F	PPHP		X		1		X	X	X	X	4	
**41**	F	PPHP		X		1		X	X	X	X	4	
**42**	M	PPHP		X		1		X	X		X	3	
**43**	F	PPHP		X		1		X		X	X	3	
**44**	F	PPHP		X		1	X	X	X	X	X	5	
**45**	M	PPHP		X		1		X	X			2	
**46**	M	PPHP		X		1		X	X	X	X	4	
**47**	F	PPHP		X		1		X	X	X		3	
**48**	F	PPHP		X		1	X	X	X			3	
**49**	F	PPHP		X		1	X	X	X		X	4	
**50**	M	PPHP		X		1		X	X		X	3	
**51**	F	PPHP		X		1		X		X	X	3	
**52**	F	PPHP		X		1		X		X	X	3	

**53**	F	PPHP		X	X	2				X		1	hyperinsulinemia, endometriosis
**54**	M	PPHP		X		1			X	X		2	
**55**	F	PPHP		X		1			X	X	X	3	PCO, hyperinsulinism, strie rubrae, acanthosis nigricans
**56**	F	PPHP		X		1	X			X		2	
**57**	F	PPHP		X		1			X	X	X	3	
**58**	F	PPHP		X		1			X		X	2	placental detachment, IUGR, hypertrichosis, ogival palate, mesomelic dysplasia, limb discomfort, adductus, nystagmus, varus knees, valgus elbows, glaucoma, dental defects, premature pubarca, dysmenorrhea, autoimmune thyroiditis, GH deficiency, Leri-Weill's discondrosteosis
**59**	F	PPHP		X		1			X		X	2	scoliosis, skeletal dysplasia, joint laxity
**60**	M	PPHP		X		1	X		X			2	
**61**	M	PPHP		X		1				X	X	2	
**62**	F	PPHP		X		1			X	X	X	3	
**63**	F	PPHP		X		1	X		X			2	
**64**	M	PPHP		X		1				X	X	2	
**65**	F	PPHP		X		1			X	X	X	3	
**66**	F	PPHP		X		1			X	X	X	3	
**67**	F	PPHP		X		1			X	X	X	3	
**68**	M	PPHP		X		1				X	X	2	
**69**	F	PPHP		X		1	X		X	X		3	
**70**	F	PPHP		X		1	X		X	X	X	4	
**71**	M	PPHP		X		1			X	X		2	
**72**	F	PPHP		X		1			X			1	
**73**	M	PPHP		X		1	X		X	X	X	4	

Supplementary table 1. Table showing the extension of 2q37 deletions found in our patients according to analyzed variable number tandem repeats (VNTRs), MLPA probes (P264-Human Telomere-9, MRC Holland) and genes location (reference assembly GRCh37/hg19); genes included in the smallest region of overlap (SRO) are in Italics. Abbreviations: HET, heterozygous; HOMO, homozygous; DEL, deleted; NO DEL, no deletion. Color legend: red, region confirmed as deleted; green, region confirmed as not deleted; yellow, region hosting the breakpoint.

gene/marker/probe ID	type	start	end	PT 1	PT 2	PT 3	PT 4
D2S206	VNTR	233.707.826	233.707.976	HET	HET	HET	HET
D2S2205	VNTR	234.400.231	234.400.401	HET	HET	HET	HET
D2S336	VNTR	235.777.094	235.777.202	HOMO	HET	HOMO	HET
D2S2202	VNTR	236.630.048	236.630.280	HOMO	HOMO	HOMO	HET
D2S338	VNTR	237.235.412	237.235.700	HOMO	HET	HET	HET
RDC1	gene	237.476.430	237.491.001				
D2S345	VNTR	237.802.011	237.802.265	HOMO	HET	HET	HOMO
D2S2968	VNTR	238.078.465	238.078.645	HOMO	HOMO	HOMO	HOMO
COL6A3/09038-L09292	gene/MLPA	238.296.676	238.296.745	DEL	NO DEL	NO DEL	NO DEL
LRRFIP1/09037-L09291	gene/MLPA	238.672.488	238.672.548	DEL	NO DEL	NO DEL	NO DEL
D2S1833	VNTR	238.690.825	238.690.951	HOMO	HOMO	HOMO	HOMO
RBM44	gene	238.707.032	238.751.451				
RAMP1	gene	238.767.536	238.820.756				
UBE2F	gene	238.875.469	238.951.236				
SCLY	gene	238.969.530	239.008.054				
ESPNL	gene	239.008.798	239.041.928				
KLHL30	gene	239.047.363	239.061.588				
FAM132B	gene	239.067.623	239.077.541				
D2S2338	VNTR	238.849.991	238.850.157	HOMO	HET	HET	HOMO
ILKAP	gene	239.079.042	239.112.370				
HES6	gene	239.146.908	239.149.303				
PER2	gene	239.152.679	239.198.743				
TRAF3IP1	gene	239.229.082	239.309.541				
TRAF3IP1/09036-L13880	gene/MLPA	239.306.132	239.306.198	DEL	NO DEL	DEL	DEL
ASB1	gene	239.335.383	239.360.891				
TWIST2	gene	239.756.673	239.795.893				
HDAC4/10036-L11449	gene/MLPA	240.274.425	240.274.494	DEL	DEL	DEL	DEL

***NDUFA10*/09034-L09288**	**gene/MLPA**	**240.960.582**	**240.960.651**	**DEL**	**DEL**	**DEL**	**DEL**
OR6B2	**gene**	**240.968.841**	**240.969.906**				
PRR21	**gene**	**240.981.230**	**240.982.399**				
OR6B3	**gene**	**240.984.494**	**240.985.489**				
MYEOV2	**gene**	**241.065.980**	**241.076.224**				
OTOS	**gene**	**241.078.446**	**241.083.979**				
**D2S125**	**VNTR**	**241.168.126**	**241.168.221**	**HOMO**	**HOMO**	**HOMO**	**HOMO**
GPC1	**gene**	**241.375.088**	**241.407.493**				
ANKMY1	**gene**	**241.418.839**	**241.508.626**				
DUSP28	**gene**	**241.499.471**	**241.503.431**				
RNPEPL1	**gene**	**241.505.221**	**241.520.789**				
***CAPN10*/15667-L17633**	**gene/MLPA**	**241.530.376**	**241.530.437**	**DEL**	**DEL**	**DEL**	**DEL**
GPR35	**gene**	**241.544.848**	**241.570.676**				
AQP12B	**gene**	**241.615.835**	**241.622.323**				
AQP12A	**gene**	**241.631.262**	**241.637.900**				
KIF1A	**gene**	**241.653.183**	**241.759.725**				
***AGXT*/10035-L11450**	**gene/MLPA**	**241.814.554**	**241.814.618**	**DEL**	**DEL**	**DEL**	**DEL**
SNED1	**gene**	**241.938.255**	**242.034.983**				
**D2S2890**	**VNTR**	**242.026.549**	**242.026.698**	**HOMO**	**HOMO**	**HOMO**	**HOMO**
**D2S1611E**	**VNTR**	**242.026.692**	**242.026.776**	**HOMO**	**HOMO**	**HOMO**	**HOMO**
MTERFD2	**gene**	**242.011.584**	**242.041.747**				
***PASK*/09031-L09285**	**gene/MLPA**	**242.080.099**	**242.080.171**	**DEL**	**DEL**	**DEL**	**DEL**
**RH25358**	**VNTR**	**242045824**	**242.045.969**	**HOMO**	**HOMO**	**HOMO**	**HOMO**
PPP1R7	**gene**	**242.088.991**	**242.123.067**				
ANO7	**gene**	**242.127.924**	**242.164.792**				
HDLBP	**gene**	**242.166.679**	**242.256.476**				
**D2S1516E**	**VNTR**	**242.168.683**	**242.168.763**	**HOMO**	**HOMO**	**HOMO**	**HOMO**
**RH101**	**VNTR**	**242.168.726**	**242.168.976**	**HOMO**	**HOMO**	**HOMO**	**HOMO**
**RH104464**	**VNTR**	**242.177.515**	**242.177.649**	**HOMO**	**HOMO**	**HOMO**	**HOMO**
NEDD5	**gene**	**242.254.515**	**242.293.442**				
FARP2	**gene**	**242.295.658**	**242.434.256**				
**RH19982**	**VNTR**	**242.384.394**	**242.384.560**	**HOMO**	**HOMO**	**HOMO**	**HOMO**

SHGC-32276	VNTR	242.418.120	242.418.223	HOMO	HOMO	HOMO	HOMO
STK25/10034-L10544	gene/MLPA	242.438.755	242.438.820	DEL	DEL	DEL	DEL
BOK	gene	242.498.136	242.513.546				
THAP4	gene	242.523.820	242.576.864				
ATG4B/02782-L02224	gene/MLPA	242.606.067	242.606.128	DEL	DEL	DEL	DEL
DTYMK	gene	242.615.157	242.626.406				
ING5	gene	242.641.450	242.668.893				
D2HGDH/15666-L17632	gene/MLPA	242.684.218	242.684.272	DEL	DEL	DEL	DEL
GAL3ST2	gene	242.716.240	242.743.623				
NEU4/09029-L09283	gene/MLPA	242.758.251	242.758.314	DEL	DEL	DEL	DEL
PDCD1/15664-L17629	gene/MLPA	242.793.165	242.793.217	DEL	DEL	DEL	DEL
CXXC11	gene	242.811.752	242.815.975				
D2S2985	VNTR	242.855.712	242.855.894	HOMO	HOMO	HET	HOMO
D2S2988	VNTR	242.856.375	242.856.543	HOMO	HOMO	HOMO	HOMO
D2S2986	VNTR	242.866.489	242.866.638	HOMO	HOMO	HOMO	HOMO
D2S447	VNTR	242.884.802	242.885.024	HOMO	HOMO	HOMO	HOMO
D2S2585	VNTR	242.926.377	242.926.556	HOMO	HOMO	HOMO	HOMO
CICP9	gene	243.062.007	243.062.204				
ABC6	gene	243.160.372	243.160.825				
chromosome end			243.199.373				
